# Supplementary material for: Cysteine Residues in Region 6 of the Plasmodium yoelii Erythrocyte-Binding-like Ligand That Are Related to Its Localization and the Course of Infection
Source: Biomolecules. 2023 Mar 2;13(3):458. doi: 10.3390/biom13030458 (PMC10046610; doi:10.3390/biom13030458)
Supplement: Supplementary file 1 [file biomolecules-13-00458-s001.zip › biomolecules-2204456-supplementary.pdf]

Table S1. Oligonucleotide primers used to generate transfection constructs

position	Primer sequence**
C717A	5' aatataataatgctagccaacgaaaatgtaGCcaatggcaatccaggtttaa 3'
	5' taaacctggattgccattgGCtacattttcgttggctagcattattatatt 3'
C726A	5' gtatgcaatggcaatccaggtttaaaatacGCtgaatctataaataataaatattttcatgga 3'
	5' tccatgaaaatatttattatttatagattcaGCgtattttaaacctggattgccattgcatac 3'
C738A	5' atctataaataataaatattttcatggaacgGCttcaaaaagtcaaacccaaaatctttgttgc 3'
	5' gcaacaaagattttgggtttgactttttgaaGCcgttccatgaaaatatttattatttatagat 3'
C747A	5' tggaacgtgttcaaaaagtcaaacccaaaatcttGCttgctcaatttcaaatta 3'
	5' taatttgaaattgagcaaGCaagattttgggtttgactttttgaacacgttcca 3'
C748A	5' gtgttcaaaaagtcaaacccaaaatctttgtGCctcaatttcaaattattgcataaaatttttt 3'
	5' aaaaaattttatGCaataatttgaaattgaggcacaaagattttgggtttgactttttgaacac 3'
C754A	5' tcaaacccaaaatctttgttgctcaatttcaaattatGCcataaaatttttttaatataaattcaaagaaatattatg 3'
	cataatatttctttgaatttatattaaaaaattttatgGCataatttgaaattgagcaacaaagattttgggtttga 3'
C754R *	5' caaattatCGCataaaattttttaata 3'
	5' ttatGCGataatttgaaattgagca 3'
C754C *	5' attatTGTataaaatttttttaatata 3'
	5' attttatACAataatttgaaattgag 3'
C768A	5' cataaaatttttaatataaattcaaagaaatattatgatGCtatgaatgaagagtttatggccccagatt 3'
	5' aatctggggccataaactcttcattcataGCatcataatatttctttgaatttatattaaaaaattttatg 3'
C780A	5' agagtttatggccccagattataacGCttttcaaaaagagagctattcaaat 3'
	5' atttgaatagctctctttttgaaaaGCgttataatctggggccataaactct 3'

* primers designed for PrimeStar® Mutagenesis Basal Kit.
** Capital letters indicate nucleotides for which the substitution is intended.

Table S2. Results of the transfection to modify *pyebl* gene locus

Cys position (amino acid position)	Substitution	Result of the transfection
Cys^{1st} (717)	C717A	success
Cys^{2nd} (726)	C726A	success
Cys^{3rd} (738)	C738A	success
Cys^{4th} (747)	C747A	success
Cys^{5th} (748)	C748A	success
Cys^{6th} (754)	C754A	failure
	C754R	failure
	C754C (synonymous)	success
Cys^{7th} (768)	C768A	success
Cys^{8th} (780)	C780A	success

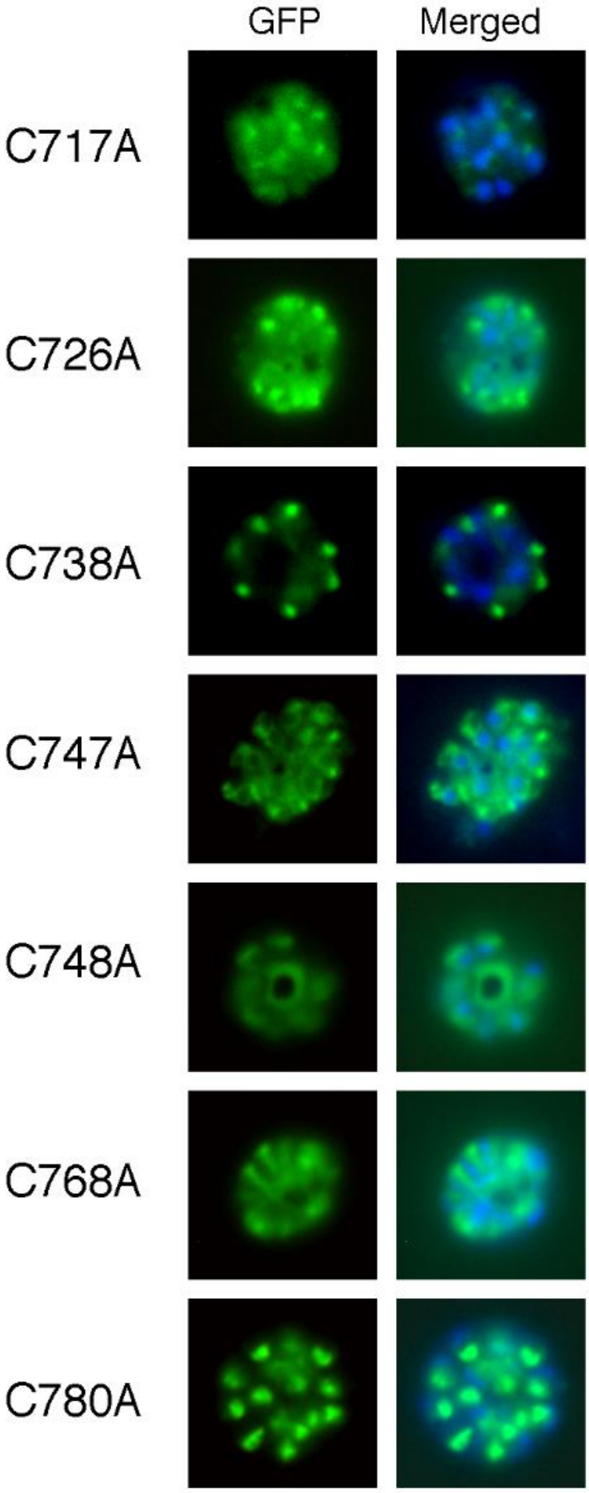

Figure S1. GFP signal of live transgenic *P. yoelii* mature schizont under fluorescent microscope. Green indicates GFP signal. Blue indicates nucleus staining.

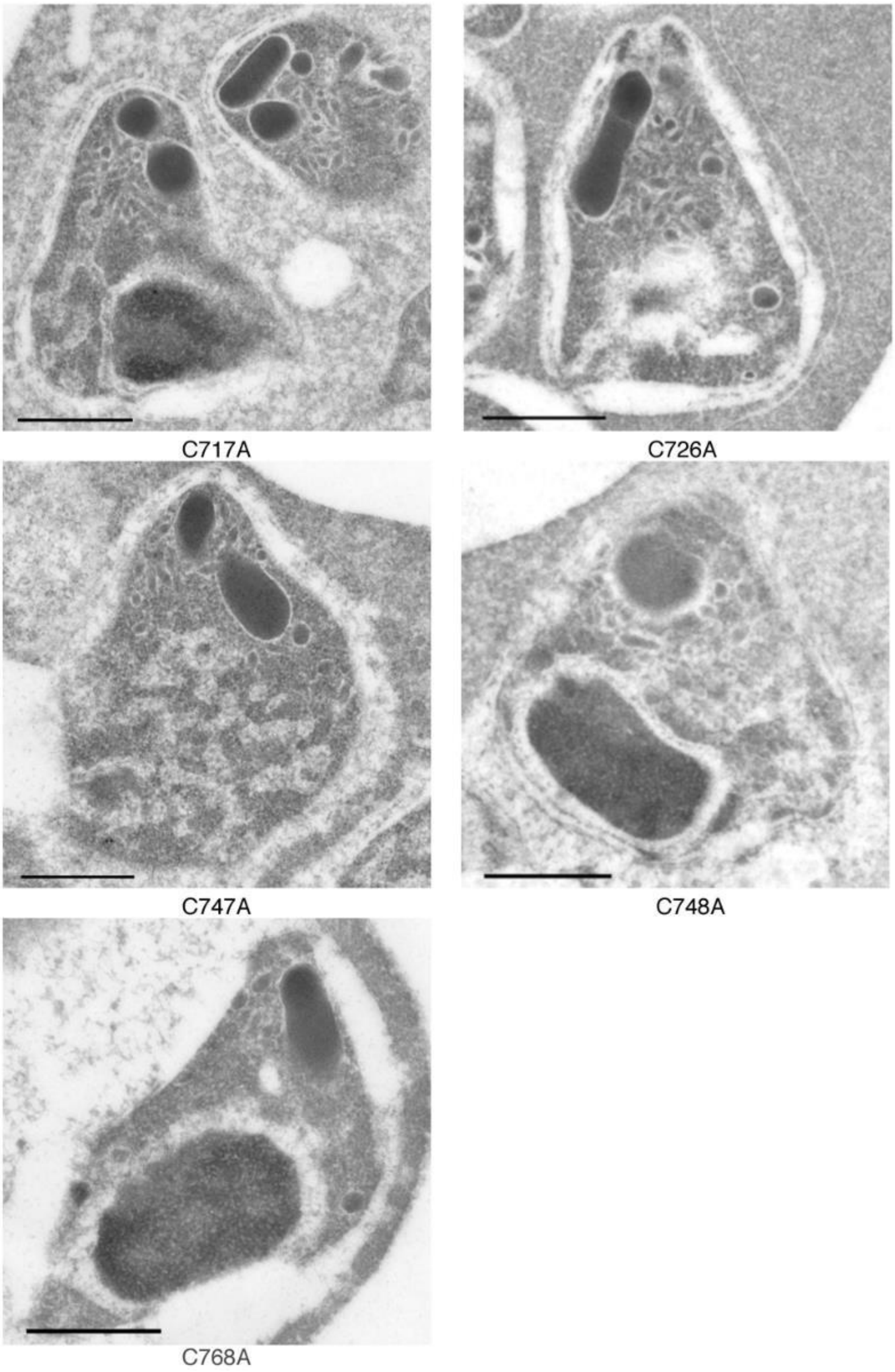

Figure S2. Immunoelectron microscopy of mature merozoites in schizonts obtained from *P. yoelii* transgenic parasite lines, C717A, C726A, C747A, C748A and C768A, showing no gold particles. The scale bars indicate 500 nm.

Material and Method S1

Production of plasmids

pPbDT3U- B12 and pHDEF1-mh-R12 were constructed as follows:

A DNA fragment encoding a cyan fluorescent protein was PCR-amplified from pECFP-C1 plasmid (Stratagene) using KOD Plus DNA polymerase with primers 5'-agcGCTAGCGTGAG-CAAGGGCGAG-3' and 5'-gacGTCGACGGATCCTCTAGACTTGTACAGCTCGTCC-3' and ligated into the pGEM-T Easy plasmid. The insert was then digested with NheI and SalI, purified, and ligated into pRGDT-B12 using the NheI and SalI sites, yielding pRCDT-B12. pRCDT-B12 was digested with ClaI and XbaI and filled with an oligonucleotide linker comprising cgatCTCGAGCCCGGGt and ctagaCCCGGGCTCGAGat to generate XhoI and SmaI sites to yield pPbDT3U-B12. pHDEF1-mh was digested with SmaI and ApaI to remove the 3' untranslated region of histidine- rich protein 2, the ApaI cohesive end was blunted, and a Gateway gene conversion cassette C1 (Invitrogen) was inserted. The XhoI site was destroyed by XhoI digestion, filled in using KOD Plus DNA polymerase, and self-ligated to yield pHDEF1-mh-R12.

Material and Method S2

Transfection and cloning

P. yoelii schizont-enriched fractions were collected by differential centrifugation on 50% HistoDenz in Tris buffer, and 25 μg of XhoI-digested transfection constructs were electroporated to 5 x 10^7 of enriched schizonts using the Nucleofector device (Amaxa) with human T cell solution under program U-33 (24). Parasites for transfection were quantified with thin Giemsa-stained smears on slide glass and hemocytometer counting. Transfected parasites were intravenously injected into 8 to 10 week-old ICR female mice. Mice were treated with 0.07 mg/ml pyrimethamine in their drinking water at 24 hours following infection. Reappearing parasites were observed by tail-blood smears on glass slides, fixed and stained with Giemsa staining; and blood was collected by cardiac puncture. Drug-resistant parasites were cloned by limiting dilution. Specifically, parasite-infected blood from donor ICR mice infected with transfected parasites was diluted such that one infected erythrocyte was injected per each mouse intravenously for 8 naïve ICR mice. Mice were checked with thin blood smears as above and infected mice were sacrificed to collect blood by cardiac puncture and preserved.

Material and Method S3

Recombinant proteins for antibody production

Wheat germ cell-free protein expression system for protein production used the bilayer translation reaction method. Briefly, 250 µl of a transcription mixture containing 25 µg of the plasmid DNA, 80 mM HEPES-KOH, pH 7.8, 16 mM magnesium acetate, 2 mM spermidine, 10 mM dithiothreitol, 2.5 mM each of nucleoside triphosphates, 250 U of SP6 RNA polymerase (Promega, Madison, WI), and 250 U of RNasin (Promega) was incubated for 6 h at 37°C. After the incubation, the transcription solution containing transcribed mRNA was mixed with 250 µl of wheat germ extract supplemented with 2 µl of 20 mg/ml creatine kinase in a single well of a six-well plate. The 5.5 ml substrate mix (30 mM HEPES-KOH, pH 7.8, 100 mM potassium acetate, 2.7 mM magnesium acetate, 0.4 mM spermidine, 2.5 mM dithiothreitol, 0.3 mM amino acid mix, 1.2 mM ATP, 0.25 mM GTP, and 16 mM creatine phosphate) from the ENDEXT Wheat Germ Expression S kit (CFS Co., Ltd., Matsuyama, Japan) was then added on top of the translation mix and incubated at 26°C for 12 h. After incubation, the reaction mixture was centrifuged at 21,900 x g for 20 min. Recovered supernatants were passed through Amicon Ultra centrifugal filter units (10kDa molecular mass cutoff) (Millipore, Billerica, MA) to replace the translation buffer with phosphate-buffered saline. The samples containing the synthesized proteins were purified using the Ni-nitrilotriacetic acid agarose column (Qiagen, Valencia, CA). Recombinant proteins were purified by passing the supernatant through the glutathione-Sepharose 4B column (GE Healthcare Bio-Sciences, Piscataway, NJ). Concentrations of affinity-purified proteins were determined using the Bradford protein assay kit (Bio Rad Laboratories, Hercules, CA). Protein samples were separated by sodium dodecyl sulfate-polyacrylamide gel electrophoresis (SDS-PAGE) under reducing conditions, and the bands were visualized with Coomassie brilliant blue for quality check.
